# Preliminary evidence for physical activity following pelvic exenteration: a pilot longitudinal cohort study

**DOI:** 10.1186/s12885-019-5860-5

**Published:** 2019-07-04

**Authors:** Daniel Steffens, Jane M. Young, Michael Solomon, Paula R. Beckenkamp, Cherry Koh, Kenneth Vuong, Matthew A. Brodie, Kim Delbaere

**Affiliations:** 1 Surgical Outcomes Research Centre (SOuRCe), Royal Prince Alfred Hospital, Camperdown, 2050 New South Wales Australia; 2 Faculty of Medicine and Health, The University of Sydney, Camperdown, NSW 2006 Australia; 3 The Institute of Academic Surgery (IAS), Royal Prince Alfred Hospital, The University of Sydney, Camperdown, 2050 New South Wales Australia; 4 Discipline of Physiotherapy, Faculty of Health Sciences, The University of Sydney, Camperdown, 2006 New South Wales Australia; 5 Falls, Balance and Injury Research Centre, Neuroscience Research Australia, University of New South Wales, 139 Barker Street, Randwick, 2031 New South Wales Australia

**Keywords:** Cancer, Physical activity, Surgery, Surgical outcomes

## Abstract

**Background:**

The physical activity (PA) level of patients undergoing major cancer surgery remains unclear. This pilot study aimed to: (i) Compare preoperative PA level between patients undergoing major cancer surgery and the general population; (ii) describe PA trajectories following major cancer surgery; (iii) Compare objective versus subjective PA measures in patients undergoing major cancer surgery; and (iv) Investigate the association between preoperative PA level and postoperative outcomes.

**Methods:**

Patients undergoing pelvic exenteration between September/2016 and September/2017 were included and followed at preoperative, 6-weeks and 6-months postoperative. PA was measured using the International Physical Activity Questionnaire Short-Form and McRoberts activity monitor. Analyses were performed using SPSS.

**Results:**

This pilot study included 16 patients. When compared to the general population, patients undergoing major cancer surgery presented a reduced preoperative PA level. PA levels decreased at 6 weeks but returned to preoperative levels at 6 months postoperative. Objective and subjective measures of PA were comparable, with some variables presenting strong correlations. A higher preoperative level PA was associated with an absence of postoperative complications and better quality of life outcomes.

**Conclusions:**

Patients undergoing major cancer surgery demonstrated lower PA levels when compared to the general population. PA trajectories decreased at 6 weeks postoperative, returning to preoperative levels within 6-months. In this cohort, it seems that higher preoperative PA level may improve postoperative surgical outcomes; however, this preliminary evidence should be confirmed in a larger cohort.

## Background

The benchmark treatment for advanced primary or recurrent cancer within the pelvis aims to completely resect all malignant disease to achieve a clear resection margin [1]. In order to accomplish this, complete or partial removal of all of the pelvic viscera, vessels, muscles, ligaments and part of the pelvic bone (ileum, ischium, pubic rami, sacrum and/or coccyx) may be required [2]. The extensive nature of this surgical procedure negatively impact on patient’s functional outcomes and quality of life in the short-term [2–4]. There is growing evidence suggesting that patients who engage in regular physical activity (PA) preoperatively, present with better postoperative surgical outcomes and quality of life [5]. Despite this, little is known about the role of PA in patients undergoing pelvic exenteration.

The health benefits of regular PA are well known, and, to achieve such benefits, the World Health Organization (WHO) recommends that adults undertake at least 150 min of moderate or 75 min of vigorous PA per week [6]. The Australian PA guidelines increased the recommendation to 300 min of moderate or 150 min of vigorous PA, in order to prevent unhealthy weight gain and to reduce the risk of non-communicable diseases, including cardiovascular disease, type 2 diabetes, anxiety, depression, musculoskeletal disorders, and some forms of cancer [7].

Recently, a systematic review [8] of 10 cohort studies demonstrated that patients with breast, colon and rectal cancer who engaged in higher levels of PA had increased survival rates. However, there is a lack of information regarding the benefits of PA for patients in the pre and postoperative period following major surgical resections for cancer [9]. To date, only a small number of studies have shown an association preoperative PA level with higher survival and lower complication rates. A prospective cohort study involving 220 patients evaluated the association between the preoperative levels of PA and recovery after breast cancer surgery. Patients who presented with higher preoperative PA levels had a faster recovery in the short-term (RR = 1.85 [95%CI = 1.20 to 2.85]) [10]. Similarly, another cohort study that included 200 patients undergoing elective cholecystectomy due to gallstone disease, investigated the effects of preoperative PA on postoperative recovery and complication rates. Regular PA participation in the preoperative period was found to be associated with better recovery and fewer complications following cholecystectomy [11].

A major limitation of these studies is the use of self-reported PA questionnaires to assess PA level. While PA questionnaires may be convenient, it has been shown that participants tend to underestimate sedentary time and overestimate PA participation when compared with data from activity monitors [12]. Another limitation of the self-reported questionnaires is recall bias, with some studies asking patients to recall their PA over the past year [13]. The most sensitive method to assess PA levels, walking bouts and sedentary time appears to be objectively, with the use of activity monitors [14, 15]. However, a direct comparison between subjective and objective PA measures has not yet been investigated in patients undergoing pelvic exenteration.

Patients facing pelvic exenteration have advanced primary or recurrent pelvic cancer with an associated heavy symptom burden. Our previous research has demonstrated low quality of life scores preoperatively compared to the general population and other surgical patient groups [3]. The aims of this study are to: (i) Compare preoperative subjective measures of PA between patients undergoing major cancer surgery and the Australian general population (ii) Describe PA trajectories among patients undergoing major cancer surgery; (iii) Compare objective versus subjective PA measures; (iv) Determine if correlations exist between objective and subjective PA measures; and (v) Investigate the association between preoperative PA level and postoperative surgical outcomes including complication rates, length of hospital stay and quality of life.

## Methods

### Study design

This study was a prospective pilot cohort that followed patients undergoing pelvic exenteration due to locally advanced primary or recurrent pelvic cancer for up to 6 months postoperatively. To reduce participant burden, this study used clinical and quality of life data already collected by the Pelvic Exenteration Surgery Quality and Improvement (PESQI) and Quality of Life in Patients with Pelvic Cancer Research Projects (Protocol No X13–0283 & HREC/13/RPAH/371 and Protocol No X16–0272 & HREC/11/RPAH/632, respectively). All included patients provided written informed consent and the Royal Prince Alfred Hospital ethics committee approved the study protocol (Protocol No X16–0327 & HREC/16/RPAH/439).

### Patients sample

A purposive sample of consecutive patients aged 18 to 80 years scheduled to undergo pelvic exenteration surgery between September 2016 and September 2017 at Royal Prince Alfred Hospital for locally advanced primary or recurrent cancer within the pelvis were invited to participate in the study. Patients were excluded from the study if they presented with: evidence of distant metastases (e.g. liver, lung, brain, bone); cognitive impairment such that they were unable to provide informed consent; a co-morbidity preventing participation in exercise (e.g. cardiac or respiratory disease); or inadequate English preventing completion of the self-reported questionnaires.

### Outcome measures

Patient demographics and clinical characteristics, PA, postoperative surgical outcomes and quality of life data were collected preoperatively, 6 weeks and 6 months postoperatively. The preoperative work-up period was used to collect the study outcome measures. This period could be from 6 to 1 week prior to their surgery.

### Subjective physical activity measure

Self-reported PA was measured using the International Physical Activity Questionnaire-Short Form (IPAQ-SF) [16]. The IPAQ-SF was used to calculate: (i) sitting time (ii) walking time; (iii) moderate PA (such as carrying light loads, bicycling at a regular pace, or tennis per day); and (iv) vigorous PA (such as heavy lifting, digging, aerobics, or fast bicycling per day), as minutes per day. Active time was calculated as the number of minutes spent on walking, moderate and vigorous physical activities per day.

The IPAQ-SF was also used to calculate the total metabolic equivalent (MET) minutes per day. Total minutes of moderate and vigorous activity per day was multiplied by a weighting (4 for moderate and 8 for vigorous activity) to calculate MET-minutes in each activity, and these values were summed to produce the total MET-minutes per day.

### Objective physical activity measure

Objective PA was measured using a small and light activity monitor comfortably attached centrally over the lower back with an elastic belt around the waist (DynaPort MoveMonitor, McRoberts, The Hague, The Netherlands) [17–19]. Participants were asked to wear the activity monitor continuously for 1 week (day and night) with the exception of activities involving immersion in water (e.g. showering). The activity monitor recorded: (i) sitting time; (ii) walking time; (iii) moderate PA (i.e., 3–6 MET-minute); and (iv) vigorous PA (i.e., > 6 MET-minute), in minutes per day. Active time was calculated as the amount of time spent on walking, moderate and vigorous physical activities per day. Total MET-minutes per day was calculated based on the duration of PA in minutes above moderate intensity (e.g. 4 MET activity for 30 min equates to 120 MET-minutes of PA).

### Measure of quality of life and patient reported outcomes

Quality of life was assessed using the Functional Assessment of Cancer Therapy – Colorectal (FACT-C) [20], and the Short Form 36 version 2 - SF36v2 [21] instruments. The FACT-C instrument provides one total score (possible range of 0 to 136). The SF-36 instrument consists of two summary scales: the physical health component scale (PCS) and the mental health component scale (MCS), each norm-based score with an average of 50 for the population and a standard deviation of 10. For both the FACT-C and SF-36 instruments, a higher score represents better QoL. Distress was measured using the Distress Thermometer [22] and pain was assessed with a study specific pain score based on pain items of the SF-36v2 instrument. Higher distress and pain scores represent worst outcomes.

### Complication rates and length of stay

Complication rates was collected within 30 days postoperative and was defined as the proportion of patients developing one or more complications according to the Clavien-Dindo classification [23], extracted from electronic medical records. Intensive care unit (ICU) and hospital length of stay was defined as the duration of inpatient hospital stay (in days) with the day of surgery considered as day 0.

### Normative physical activity data

Normative data from the general Australian population that was previously established using the IPAQ-SF in a published cohort study was used for comparison. This cohort is a nationally representative random sample of Australians with matched age range [24].

### Statistical analyses

All statistical analyses were performed using IBM SPSS Statistics version 24 (SPSS Inc., Chicago, IL, USA). Descriptive statistics were used to summarise baseline patient characteristics and postoperative surgical outcomes. Median and interquartile range (IQR) were calculated for subjective and objective PA measures at each time point.

The Mann Whitney U test was used to compare preoperative subjective PA measures between the study cohort and the normative Australian PA data.

Comparison between combined preoperative, 6 weeks and 6 months PA measures was performed using Wilcoxon’s signed rank test.

To measure the strength of association between subjective and objective measures of PA, we used the Spearman rho correlation and 95% confidence intervals. The strength of the correlation was defined as weak (0.10 to 0.39); moderate (0.40 to 0.49); or strong (0.50 to 1.0) [25].

A series of linear and logistic univariate analyses were used to assess the association between subjective and objective measures of preoperative PA and length of hospital stay, length of ICU stay, postoperative complication rates, and measures of pain, distress and quality of life at 6 weeks and 6 months postoperative. A significant *p* value was set as 0.05 for all analyses.

## Results

During the study period, 43 patients were screened for eligibility. Of these, 16 patients including 10 males, with median age of 54 years (IQR = 46 to 65), undergoing pelvic exenteration for advanced or recurrent pelvic cancer were included. The median length of hospital stay was 16 days, with two thirds presenting with at least one complication (*N* = 12, 66.7%) postoperatively (Table 1). No difference was found between the patients that consented (*N* = 16) and those who did not consent (*N* = 27) to the study, in terms of age, gender, length of hospital and ICU stay and postoperative complications (*p* > 0.05).

**Table 1:** Characteristics of the study sample (*N* = 16)

Variables	Median (IQR) or N (%)
Age (years)	54.00 (46.00 to 65.00)
Gender (male)	10 (55.60%)
Length of hospital stay (days)	16 (13.50 to 22.00)
Length of ICU stay (days)	2.50 (1.75 to 5.00)
Postoperative complication (presence)	
Sepsis	5 (27.80)
Wound	8 (44.40%)
Cardiovascular	3 (16.70%)
Gastrointestinal	5 (27.80%)
Urological	4 (22.20%)
Ostomy	7 (38.90%)
Respiratory	4 (22.20%)
Neurological	2 (11.10%)
Overall complication rate^a^	12 (66.70%)
Baseline	
Distress (*n* = 16)	1.50 (0.00 to 5.00)
Pain (*n* = 15)	2.00 (0.00 to 3.00)
Physical Component Score (SF-36), (*n* = 16)^b^	48.50 (36.50 to 54.00)
Mental Component Score (SF-36), (*n* = 16)^b^	50.50 (38.00 to 58.00)
FACT-C (*n* = 15)^c^	106.00 (95.00 to 118.00)
6 Weeks postoperative	
Distress (*n* = 12)	1.50 (0.00 to 5.00)
Pain (*n* = 13)	4.00 (1.50 to 7.00)
Physical Component Score (SF-36), (*n* = 13)^b^	38.00 (27.00 to 50.00)
Mental Component Score (SF-36), (*n* = 13)^b^	47.00 to (37.50 to 54.50)
FACT-C, (*n* = 13)^c^	81.00 (71.50 to 91.00)
6 Months postoperative	
Distress (*n* = 12)	1.50 (0.25 to 5.00)
Pain (*n* = 12)	3.50 (2.00 to 6.75)
Physical Component Score (SF-36), (*n* = 12)^b^	42.00 (38.00 to 50.50)
Mental Component Score (SF-36), (*n* = 12)^b^	49.00 (40.25 to 57.75)
FACT-C (*n* = 12)^c^	89.50 (78.75 to 100.00)

*IQR* Interquartile range

^a^Number of individual patients presenting ≥1 in hospital complication;

^b^Possible range for SF-36® physical component scale and mental component scale: norm-based scores with an average of 50 for the population and a standard deviation of 10;

^c^Possible range for FACT-C: 0–136; a higher score represents better quality of life

**Fig. 1:**
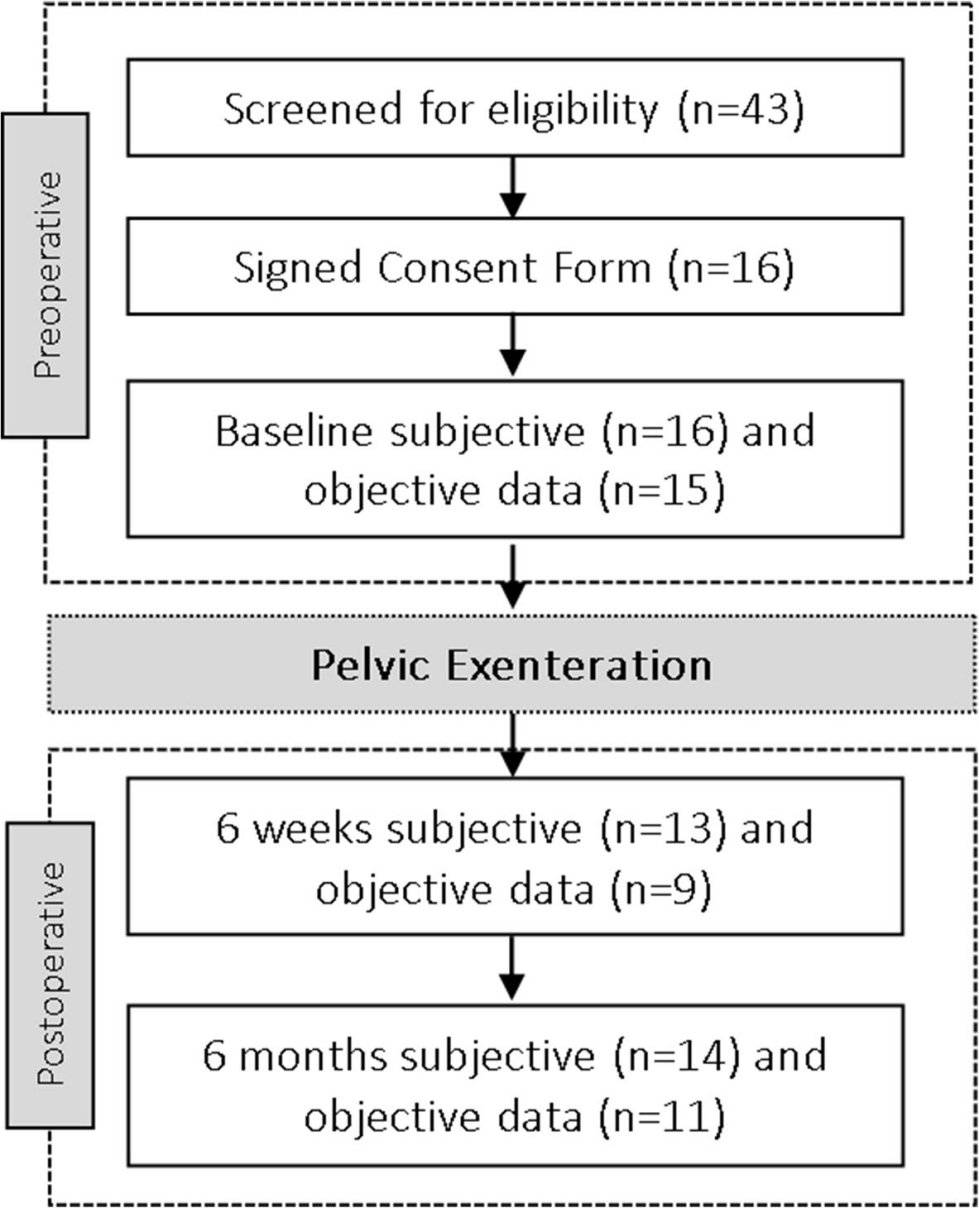
Flow of the included patients

At baseline, subjective PA data was available for all patients and objective PA data in 15 patients (94%). The number of patients with available PA data dropped over time. Subjective PA data was available for 13 (81%) and 14 patients (88%), and objective data were available for 9 (56%) and 11 (69%) patients at 6 weeks and 6 months post-surgery respectively (Fig. 1).

### Physical activity measures of pelvic exenteration patients, compared to the general population

Subjective preoperative measures of PA for patients undergoing surgery for advanced or recurrent pelvic cancer (*n* = 16) were compared to the general Australian population (*n* = 1448). Table 2 shows that the general population was significantly more active than the patients from this study, for all activity measures except time spent walking per day (*p* = 0.727).

**Table 2:** Comparison of physical activity measures between major cancer surgery patients and Australian population

Variables	Major cancer surgery patients (*n* = 16)	Australian Cohort (*n* = 1448)	*P* value
	Median (IQR)	Median (IQR)	
Sitting time (min/day)	360.00 (277.50 to 525.00)	240.00 (180.00 to 240.00)	0.039
Walking time (min/day)	18.50 (14.25 to 100.00)	30.00 (11.00 to 60.00)	0.727
Moderate activity (min/day)	0.00 (0.00 to 7.75)	13.00 (0.00 to 48.75)	0.001
Vigorous activity (min/day)	0.00 (0.00 to 5.50)	17.00 (0.00 to 60.00)	0.003
Active time (min/day)	22.00 (17.00 to 174.00)	88.00 (34.00 to 214.00)	0.021
MET-minute/per day	8.50 (0.00 to 72.75)	240.00 (57.00 to 720.00)	< 0.001

Physical activity measured with the International Physical Activity Questionnaire (IPAQ-SF)

### Physical activity trajectory

**Fig. 2:**
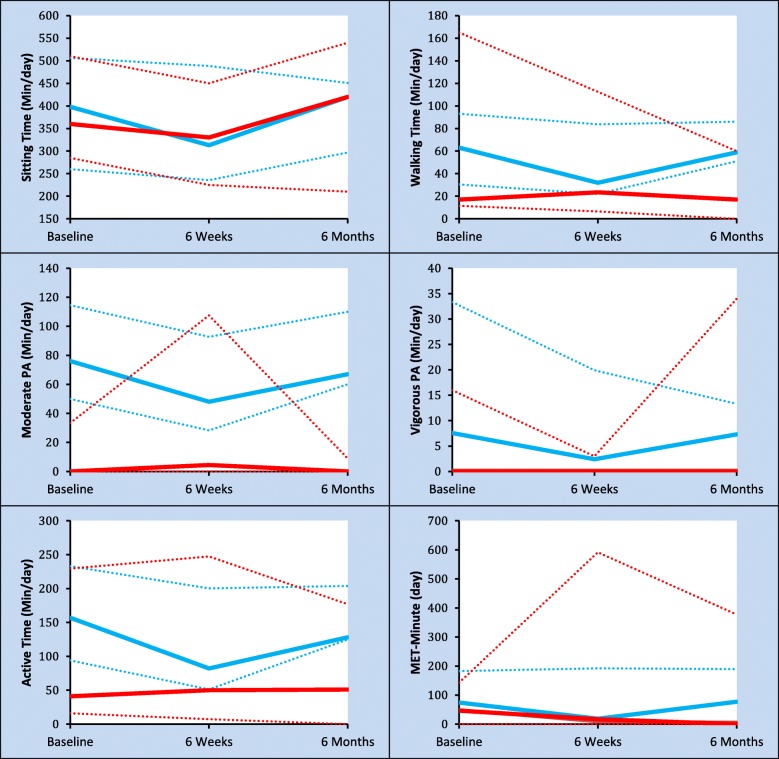
Subjective (red line) and objective (blue line) physical activity trajectories. Included patients that provided objective and subjective measures of physical activity on at least one single time point. Values are median (solid line) and interquartile range (dotted line)

Subjective and objective PA trajectories are presented in Fig. 2, from 9 patients at 6 weeks and 11 patients at 6 months for whom both objective and subjective PA data was available. Objectively measured active time decreased between baseline and 6 weeks postoperative for all measures, returning to preoperative levels within 6 months postoperative. There were no changes in the subjective measures of active time during the study period or sitting time.

### Comparison between objective and subjective physical activity measurers

**Fig. 3:**
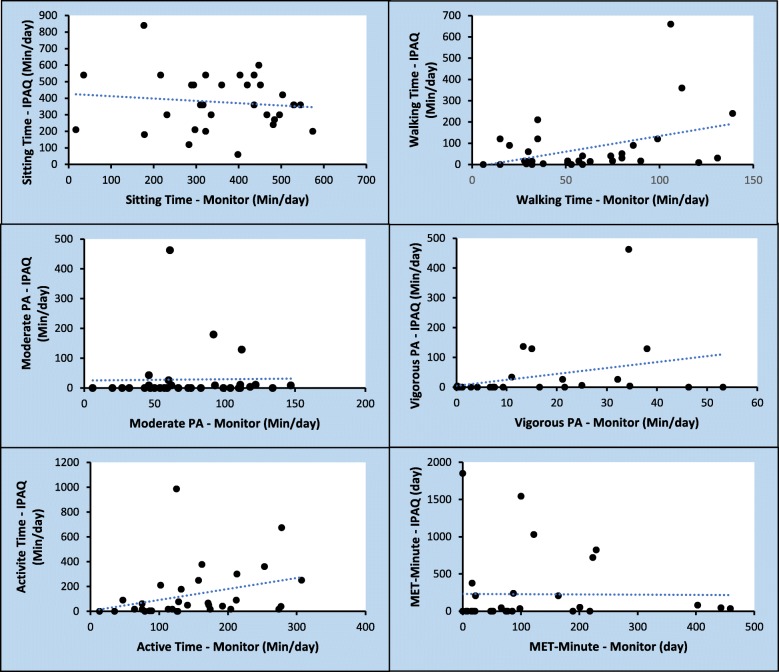
Correlation between objective (Monitor) and subjective (IPAQ) measures of physical activity

All subjective and objective measures of PA from the three time-points (baseline, 6 weeks postoperative and 6 months postoperative) were grouped and compared, resulting in a combined data pool of 32 cases. Measurements of sitting time, walking time, vigorous PA, active time and MET-minutes were similar when measured objectively with the activity monitor or subjectively with the IPAQ-SF (Table 3). PA levels of moderate activities, on the other hand, was consistently lower on the IPAQ-SF (median = 0.00; IQR = 0.00 to 9.00) when compared to the objective data (median = 67.00; IQR = 47.75 to 108.50). Similarly, a moderate to strong association was observed for walking time (*r* = 0.38; *p* = 0.033), vigorous activity (*r* = 0.51; *p* = 0.003), active time (*r* = 0.50; *p* = 0.003) and MET-minute (*r* = 0.41; *p* = 0.019). Subjective and objective measures of sitting time and moderate activity were not significantly correlated with each other (*r* = − 0.08; *p* = 0.668 and *r* = 0.27; *p* = 0.130, respectively). A graphical exploration of these relationships is show on Fig. 3.

**Table 3:** Comparison between objective (monitor) and subjective (IPAQ-SF) physical activity measures following pelvic exenteration (*n* = 32)

Variables	Objective (Monitor)	Subjective (IPAQ-SF)	*P* value^a^
	Median (IQR)	Median (IQR)	
Sitting time (min/day)	379.00 (290.00 to 477.25)	360.00 (247.50 to 480.00)	0.940
Walking time (min/day)	55.00 (30.50 to 84.50)	17.00 (9.00 to 90.00)	0.364
Moderate activity (min/day)	67.00 (47.75 to 108.50)	0.00 (0.00 to 9.00)	< 0.001
Vigorous activity (min/day)	7.00 (0.25 to 21.46)	0.00 (0.00 to 5.50)	0.126
Active time (min/day)	130.00 (86.75 to 201.00)	45.00 (15.50 to 201.75)	0.068
MET minute/per day	70.00 (15.25 to 182.75)	0.00 (0.00 to 206.00)	0.705

^a^Difference between objective (monitor) and subjective (IPAQ) PA measures (Wilcoxon’s signed rank test)

### Association between preoperative physical activity measures and postoperative outcomes

Length of hospital and ICU stay and measures of pain and psychological distress at 6 weeks and 6 months postoperative were not associated with preoperative PA levels (data not shown). Table 4 presents the significant results from a series of univariate analyses and shows that higher levels of measured active time preoperatively (especially, moderate and vigorous PA) were associated with better FACT-C scores at 6 weeks. These results were confirmed using both objective and subjective measures. In our cohort, better SF-36 physical component scores at 6 months and the absence of postoperative complications were only predicted by objectively-measured active time data.

**Table 4:** Univariate analyses reporting the association between preoperative physical activity measures with complication rates and postoperative outcomes

Variables	N	Univariate analysis		
		Beta	95% CI	*P*
FACT-C – 6 weeks postoperative				
Moderate activity (min/day), Monitor	11	0.22	0.01 to 0.42	0.040
Vigorous Activity (min/day), Monitor	11	0.59	0.19 to 0.99	0.009
Vigorous Activity (min/day), IPAQ-SF	12	0.22	0.06 to 0.38	0.012
Active time (min/day), Monitor	11	0.11	0.02 to 0.20	0.025
MET-minute/day (IPAQ-SF)	11	0.03	0.01 to 0.05	0.009
Physical Component Score (SF-36) – 6 months postoperative				
Vigorous Activity (min/day), Monitor	11	0.47	0.08 to 0.85	0.024
Variables	N	Univariate analysis		
		Odds Ratio	95% CI	*P*
Complication rates (present/ absent)				
Walking (min/day), Monitor	11	0.96	0.92 to 0.99	0.045
Moderate activity (min/day), Monitor	11	0.94	0.88 to 0.99	0.041
Vigorous Activity (min/day), Monitor	11	0.84	0.71 to 0.98	0.031

Length of hospital and ICU stay, Distress, pain, mental component score (SF-36) and the physical component score (SF-36) at 6 weeks, and distress, pain, mental component score (SF-36) physical component score (SF-36), and the Functional Assessment of Cancer Therapy-Colorectal (FACT-C) at 6 months were not reported in the table due to presenting *p* > 0.05 in the univariate analysis

## Discussion

Our study showed that patients who are about to undergo major surgery for locally advanced or recurrent cancer within the pelvis were less physically active than the general Australian population. This prospective cohort study is the first to investigate subjective and objective PA trajectories at 6 weeks and 6 months after surgery. Our results showed that PA levels decreased at 6 weeks but returned to preoperative levels at 6 months postoperative. Subjective (IPAQ-SF) and objective (activity monitor) measures of active time were mostly comparable, except for moderate PA. We also found that higher levels of vigorous PA preoperatively were significantly associated with a decrease in postoperative complications and better postoperative quality of life outcomes.

This study has a number of strengths, particularly in terms of the longitudinal nature of this study for up to 6 months postoperative. Another strength of this study is the use of validated subjective and objective measures of PA, quality of life and patient reported outcomes. The activity monitor (McRoberts) and the IPAQ-SF are widely used and well-validated objective and subjective measures in the assessment of PA, respectively [16–19]. There are some limitations of this study that should be acknowledged, most notably the small numbers and high attrition rate, which affects the representativeness of the sample. In addition, no measures of PA were collected from the patients that did not consent to the study, precluding further analysis to investigate selection bias. A consecutive sample of patients undergoing a major pelvic cancer surgery were recruited, in attempt to improve representativeness, but the seriousness of the planned surgery might have affected their motivation to participate in research. Furthermore, it is also important to note that patients undertake preoperative sessions of chemoradiotherapy, which could decrease PA participation. Despite these limitations, this study contributes to a better understanding of the potential advantages of being as physically active as possible before major surgery. Future studies should attempt to validate these findings and need to be undertaken in a larger cohort.

Interestingly, previous studies have demonstrated that subjective measures of PA (i.e. collected via questionnaires) tend to overestimate PA participation and underestimate sitting time [26]. While our study found that only moderate PA reported a significant difference between subjective and objective measures, all median values of subjective PA measures were lower than the objective measures collected with the activity monitors. Moreover, most subjective and objective PA measures presented a weak to moderate correlation with each other. If these correlations are confirmed in a larger study, it could help solve some of the issues reported when collecting objective PA measures (i.e. insufficient wearing time, malfunction and financial costs) [27].

Previous studies investigating the association between preoperative PA levels and postoperative outcomes in patients undergoing colorectal, oesophageal, prostate and breast cancer surgery [10, 11, 28, 29]. The results are conclusive that patients who were more physically active preoperatively recovered more quickly postoperatively [11] with a reduced need for sick leave when compared to less physically active patients [29]. Our study added to this literature by showing a similar trend in patients undergoing major pelvic cancer surgery and uniquely showed a promising risk reduction for suffering postoperative complications of up to 14% for every minute of PA preoperatively per day. Interestingly, higher intensity activity, such as vigorous PA (OR = 0.84; 95%CI = 0.71 to 0.98), presented a stronger association with the absence of postoperative complications than walking (OR = 0.96; 95%CI = 0.92 to 0.99) and moderate PA (OR = 0.94; 95%CI = 0.88 to 0.99).

Our study has important clinical implications. The Clinical Oncology Society of Australia (COSA) has recently released a position statement recommending cancer patients to be more physically active and reduce sedentary behaviour. The recommendations are to incorporate at least 2.5 h of moderate intensity aerobic exercise and two to three moderate intensity resistance exercise sessions each week [30]. The findings of our study support this position statement and also informs dose and intensity of preoperative exercise programs to potentially reduce the risk for postoperative complications and improve health-related quality of life outcomes at 6 weeks and 6 months postoperative. Our results suggest that high intensity exercise programs may provide a greater benefit to these patients, especially considering their already lower PA levels and extended sitting time when compared to the general population [24]. However, the small sample size of our study precluded the inclusion of age and preoperative comorbidities as covariates and, therefore, an appropriately powered randomized control trial is now required to determine if an exercise intervention might be a cost-effective way to increase preoperative PA and to determine efficacy to improve postoperative outcomes in people undergoing major cancer surgery. Moreover, future studies should investigate the potential association between preoperative PA level and overall health status. The influence of comorbidities should also be investigated.

## Conclusions

This study found that PA levels for patients undergoing major cancer surgery decline at 6 weeks postoperative, retuning to preoperative levels within 6 months postoperative. We also, found that subjective and objective measures of PA are comparable, with some components of PA presenting weak to moderate correlations. Furthermore, a higher preoperative level of vigorous PA was associated with an absence of postoperative complications and better quality of life outcomes. When compared to normative values of PA, patients undergoing major cancer surgery presented a reduced level of PA. Due to the small sample of patients included in this prospective cohort study, these findings must be confirmed in a larger study and therefore caution should be taken when interpreting the results.

## Data Availability

The datasets used and/or analysed during the current study available from the corresponding author on reasonable request.

## Abbreviations

COSA: Clinical Oncology Society of Australia
FACT-C: Functional Assessment of Cancer Therapy-Colorectal
ICU: Intensive Care Unit
IPAQ-SF: International Physical Activity Questionnaire – Short Form
IQR: Interquartile range
MCS: Mental Component Score
MET: Metabolic Equivalent
OR: Odds ratio
PA: Physical Activity
PCS: Physical Component Score
PESQI: Pelvic Exenteration Surgery Quality and Improvement
QoL: Quality of life
SF-36: Short from 36
WHO: World health organisation
